# Editorial: Soybean breeding for abiotic stress tolerance: towards sustainable agriculture

**DOI:** 10.3389/fpls.2025.1578039

**Published:** 2025-04-25

**Authors:** Huatao Chen, Li Song, Henry T. Nguyen, Donghe Xu, Chengfu Su

**Affiliations:** ^1^ Institute of Industrial Crops, Jiangsu Academy of Agricultural Sciences, Nanjing, China; ^2^ College of Horticulture, Yangzhou University, Yangzhou, China; ^3^ Division of Plant Sciences, University of Missouri, Columbia, SC, United States; ^4^ Biological Resources and Post-harvest Division, Japan International Research Center for Agricultural Sciences (JIRCAS), Tsukuba, Japan; ^5^ College of Agronomy, Qingdao Agricultural University, Qingdao, China

**Keywords:** soybean, abiotic stress, tolerance, KASP, biotechnology

Soybean, with its high - protein and oil - rich seeds, is a vital agricultural commodity. However, the growing global demand, spurred by population growth and dietary changes, poses significant challenges in meeting production demand. Abiotic and biotic stresses, such as drought, salinity, extreme temperatures, and flooding, are major hurdles in soybean breeding.

To address these challenges, modern soybean breeding strategies aim to develop stress tolerant varieties. Advancements in molecular biology and biotechnology provide innovative approaches to this end. Techniques such as Kompetitive Allele-Specific PCR (KASP) molecular markers help breeders to accurately select stress - tolerant genes, while omics - based approaches provide insights into the molecular mechanisms underlying soybean’s stress responses. These findings are presented in eleven papers on the Research Topic “Soybean Breeding for Abiotic Stress Tolerance: Towards Sustainable Agriculture.”

## Salt tolerance

Wang et al. investigate the genetic basis of salt tolerance in soybeans, a crucial aspect considering the global issue of soil salinization. A natural population of 283 soybean germplasms was used for this study. After identifying 180 mM NaCl as the optimal stress concentration, germination traits such as germination rate, energy, and index were measured under salt stress conditions. Through a genome-wide association study (GWAS), 1841 significant SNPs associated with these traits were identified, leading to the identification of 12 candidate genes. KASP markers were developed for specific SNPs. These findings offer valuable insights into soybean salt tolerance and offer genetic resources and a theoretical foundation for breeding salt-tolerant soybean varieties.

Xu et al. aimed to identify salt-tolerance-associated SNPs in soybean and evaluate genomic prediction for salt tolerance, using 563 germplasms from twenty-six countries. They identified four subpopulations (Q1-Q4) through relevant analyses. GWAS identified 10 SNPs on chromosomes 1, 2, 3, 7, and 16 that were significantly associated with salt tolerance, with eleven candidate genes located near 7 of these SNPs. Genomic prediction models including maBLUP, gBLUP, and sBLUP showed moderate - to - high r - values, and the GWAS-derived SNP marker set was effective for genomic selection. The genetic diversity analysis of salt-tolerant germplasms indicated a broad genetic background and highlighted the influence of geographic factors on salt tolerance in soybeans.

## Drought tolerance

In response to the challenge of drought affecting soybean production in China, Jia et al. focused on a natural population of 264 Chinese soybean accessions. They treated these accessions with 15% PEG - 6000 during germination and employed a series of evaluation indexes, identified 17 drought - tolerant germplasms. Utilizing Genome - Wide Association Studies (GWAS), 92 SNPs and 9 candidate genes related to drought tolerance were discovered. Moreover, two KASP markers associated with drought tolerance were developed, which not only augment the soybean germplasm pool but also establish a crucial basis for molecular breeding of drought-resistant soybean varieties.

## Flooding tolerance

Soybean is highly sensitive to flooding stress, and the Hypoxia Inducible Gene Domain (*HIGD*) gene family may play a role in plant responses to hypoxia. Geng et al. identified six *GmHIGD* genes in the soybean genome. The genes have conserved genomic structures and motifs. Chromosomal location and collinearity analysis revealed their distribution and potential evolutionary relationships. Cis - element analysis of promoters and TF identification suggested their involvement in growth, development, and stress responses. Expression analyses across various tissues and stresses (flooding, hypoxia, drought, salt) were conducted. Notably, GmHIGD3 was found to be localized in mitochondria, and its overexpression in *Arabidopsis* affected catalase activity and ROS content. Overall, this research provides valuable insights into the characteristics and potential functions of the *GmHIGD* gene family in soybean.

## Shoot tolerance

Jia et al. investigate the impact of shade on soybean yield, with a focus on identifying shade-tolerant genomic loci and varieties. A natural population of 264 soybean accessions was subjected to a 30% light reduction treatment. GWAS was conducted on six agronomic traits and shade tolerance coefficients (STCs). Five high shade-tolerant germplasms were found, and a total of 733 significant SNPs associated with STCs of six traits were detected over two years. Four candidate genes related to shade tolerance were found. Additionally, KASP markers were developed for four SNPs, and haplotype analysis was performed. These results provide valuable genetic resources and new insights for soybean shade tolerance breeding and theoretical research.

## Root architecture

In soybean, root architecture traits are vital for plant performance. The root length locus qRL16.1 on chromosome 16 has been previously reported. Through transcriptome analysis of near - isogenic lines (NILs), Kumawat et al. characterized two candidate genes, *Glyma.16g108500* and *Glyma.16g108700*, which exhibited higher expression in longer root accessions. The C-terminal domains of these genes are similar to those of C-terminally encoded peptides (CEPs) in *Arabidopsis*, known to regulate root length and nutrient response. Two polymorphisms located upstream of *Glyma.16g108500* were associated with root length traits. Synthetic peptide assays showed a positive effect of the predicted CEP variants on primary root length. These genes are specifically expressed in the root during the early growth stage and shown differential expression pattern only in the primary root. They hold potential for improving soybean to develop a strong root system under low moisture and nutrient conditions.

## Seed traits

Soybean seed viability is crucial for both quality and production yet seed quality and germplasm preservation face challenges. Li et al. investigated the mechanisms of soybean seed aging by using aging-sensitive R31 and aging-tolerant R80 lines, subjecting them to artificial aging treatments of varying durations. Analyses of the transcriptome and metabolome revealed that the response to aging stress is associated with the phenylpropanoid metabolism pathway, in which caffeic acid plays a key role. Furthermore, soaking seeds in caffeic acid was found to enhance germination rates. These fundings provide a theoretical basis for future research on soybean seed aging mechanisms.

## RNA modification

Despite the known significance of N6 - methyladenosine (m6A) RNA modification in regulating biological processes, its genome - wide identification and functional characterization in legumes like soybean have been lacking. Liu et al. used bioinformatics to identify thirteen m6A writer complex genes in soybean, which grouped into four families. They analyzed the characteristics, enzymatic activities, and expression patterns of these genes under abiotic stresses conditions, highlighting the roles of GmMTAs and GmMTBs in soybean’s response to abiotic stress. This study establishes a foundation for further exploration of the functions of m6A modification in soybean.

